# Diagnostic accuracy of a Brix refractometer to estimate porcine colostrum quality

**DOI:** 10.1002/vetr.5666

**Published:** 2025-08-21

**Authors:** Áine C. O'Brien, Marianne Farish, Emma M. Baxter, Katharine S. Denholm

**Affiliations:** ^1^ School of Biodiversity, One Health and Veterinary Medicine University of Glasgow Glasgow UK; ^2^ Animal and Veterinary Sciences Scotland's Rural College Edinburgh UK

**Keywords:** Brix, colostrum, evaluation, immunoglobulin G, pen‐side test, pigs

## Abstract

**Background:**

The objective of this study was to investigate the validity of the Brix refractometer to accurately estimate immunoglobulin G (IgG) concentrations in porcine colostrum, using radial immunodiffusion (RID) as a reference standard.

**Methods:**

Eighty‐seven composite colostrum samples were collected from sows on a single pig farm between September 2022 and September 2024. Brix measurements were compared with IgG RID test results for each colostrum sample.

**Results:**

Brix measurements ranged from 10.80% to 28.05% (mean = 20.13; standard deviation = 3.78). Samples from 40 of the 87 sows (45.98%) were below the colostrum quality threshold of 50 g/L IgG. Pearson's correlation coefficient revealed a strong correlation of 0.81 (*p* < 0.001) between Brix (%) and RID IgG concentration. Receiver operating characteristic (ROC) curves were constructed to determine the optimal threshold to accurately determine poor colostrum quality (<50 g/L IgG) using the Brix refractometer. At an RID IgG concentration threshold of less than 50 g/L, ROC analysis (Youden index) defined an optimal Brix threshold of 20.05% (sensitivity = 0.75, specificity = 0.75 and area under the curve = 0.76).

**Limitations:**

The retrospective design of this study led to some inherent limitations with the quality and quantity of the data collected.

**Conclusions:**

The results of this study indicate that the Brix refractometer can be used as an accurate pen‐side tool for estimating IgG concentrations in porcine colostrum.

## INTRODUCTION

The epitheliochorial nature of the porcine placenta means that piglets rely on the ingestion of colostrum immediately after birth to acquire maternally derived antibodies for passive immunity until their own immune system develops.^1^ The intake of an adequate volume of good‐quality colostrum within a short period after farrowing is critical to the health and future productive performance of neonatal piglets.^2^ Piglets are said to suffer from failure to transfer passive immunity (FTPI) when this process is suboptimal.^2^

While the parameters of colostrum management are widely acknowledged in dairy calves (calves should receive 10‒12% of their bodyweight in colostrum containing ≥50 g/L IgG [22% Brix] within 6 hours of birth^3^), the same defined parameters have not yet been established for piglets, since they are usually left to suckle their dams, unlike dairy calves that are typically removed from their mothers within 24 hours after birth.^3^ The quantity of colostrum consumed by piglets during the first 24 hours after birth can vary considerably, and insufficient colostrum intake is one of the major causes of piglet mortality.^4^, ^5^, ^6^ Studies have reported an average colostrum intake of 250–300 g/day.^7^, ^8^ However, more recently, Quesnel et al. reported colostrum intakes averaging 456 g/day, although there was a large range (from 56 to 1034 g/day).^6^ It is estimated that a minimum of 200 g of liquid colostrum per piglet is required within 24 hours of birth to reduce pre‐weaning mortality, provide sufficient passive immunity and promote weight gain.^2^ Furthermore, Quesnel et al.^2^ recommended that each piglet should consume 250 g of colostrum, equating to 180 g/kg birth weight, given that the average piglet birth weight (in their study) was 1.4 kg. This equates to piglets consuming 17.85% of their birth weight in colostrum.

Due to welfare, production and economic implications, pre‐weaning piglet mortality remains a considerable concern to the pig industry. Pre‐weaning piglet mortality has been reported to range from 10% to 24% in the main pig‐producing countries.^9^, ^10^, ^11^ The average pre‐weaning piglet mortalities in the UK and the EU are 12.6% and 14%, respectively.^12^ Most deaths in the pre‐weaning period occur within the first 72 hours after birth.^11^

The colostrum IgG concentration varies greatly between sows,^8^, ^13^ even within the same unit and management regime.^14^ Risk factors such as sow parity, exogenous hormones, genotype, nutritional status, vaccination status and feed fat content are reported to influence colostrum composition and IgG concentration,^7^, ^8^ as well as environmental stressors (e.g., thermal stress).^15^, ^16^ While colostrum production is reportedly independent of litter size,^7^, ^8^ the longer farrowing duration of highly prolific sows has been identified as a risk factor for reduced sow colostrum yield.^5^, ^17^ Furthermore, larger litter sizes with more live‐born piglets resulted in a lower colostrum intake per piglet.^7^, ^18^, ^19^ This is linked to greater competition at the udder, as well as a higher propensity of low birth weight and low‐viability offspring per litter, affecting their ability to compete.^10^

In addition to crucial immune transfer from the dam, sufficient, timely intake of good‐quality colostrum provides energy for thermoregulation^1^ and stimulates the development and functional maturation of the piglets’ intestines,^20^ promoting average daily gain (ADG). The literature suggests an achievable ADG for pre‐weaning piglets to be 205‒220 g/day,^21^, ^22^ but the target ADG for piglets is not well established in the peer‐reviewed literature. The volume of colostrum consumed by piglets within the first 24 hours of birth has considerable long‐term benefits for piglet growth. For example, Devillers et al. reported that piglets that consumed more than 290 g of colostrum within the first 24 hours of birth gained approximately 2 kg more in bodyweight by 6 weeks of age than those that consumed less than 290 g of colostrum.^23^

Colostrum quality (i.e., IgG concentration) can be measured directly using radial immunodiffusion (RID) or enzyme‐linked immunosorbent assay (ELISA). ELISA is currently not available in Scottish commercial laboratories, and RID is expensive and technically demanding. Furthermore, both ELISA and RID are not practical for immediate colostrum analysis, as both require laboratory settings and considerable turnaround times. Optical or digital Brix refractometry enables cost‐effective, indirect, pen‐side estimation of colostrum and can be done immediately on‐farm. The Brix refractometer measures the total solids in a liquid through refraction of light, which indirectly estimates the concentration of IgG in colostrum.^24^ On‐farm evaluation of colostrum IgG content using a Brix refractometer is a practice routinely used in sheep and goats,^25^ dairy cattle^26^ and horses.^27^ This practice is currently not routinely used in the pig industry but has the same potential to enable the estimation of IgG concentration in both sow colostrum and piglet serum to monitor the success of passive transfer in neonatal piglets.^28^

The objective of the present study was to evaluate the diagnostic accuracy of the Brix refractometer to estimate porcine colostrum quality and define thresholds for IgG concentration in porcine colostrum (using RID as the reference standard).

## MATERIALS AND METHODS

### Sample size calculation

A minimum sample size of 77 colostrum samples was required based on conservative receiver operating characteristic (ROC) curve area under the curve (AUC) estimates of 0.85, a type 1 error of 0.05 and a power of 0.9 with a 10% positive sample prevalence (MedCalc Software, version 22.018).

### Animal data

Eighty‐seven stored colostrum samples (frozen at ‒80°C) and retrospective farrowing and pre‐weaning piglet data were conveniently available from the research pig unit of Scotland's Rural College (SRUC), Midlothian, Scotland. Colostrum samples were collected from gilts and sows (parity range 1‒12, mean = 2.72) between September 2022 and September 2024 and analysed retrospectively from stored samples. The primary sow breed was Landrace X Large White (Pig Improvement Company), while the sire breed of the litters was Large White for damline replacement litters (35 litters) or Danish Duroc for terminal litters (52 litters). Sow data pertinent to this study included farrowing group, date of artificial insemination, date and time of farrowing, gestation length, litter size and date/time of colostrum collection.

### Sample collection

One trained technician collected composite colostrum samples manually from sows lying laterally when suckling their litter. Teats were not wiped prior to sample collection to mimic the colostrum the piglets were ingesting. The technician sampled, at random, from at least one of each of the top, middle and bottom teats (without pre‐stripping). When possible, colostrum samples were collected within 6 hours postpartum, after most piglets would have suckled at least once. Sow farrowing was supervised by a trained stockperson between 8 am and 5 pm daily, but was unsupervised outside these hours. For the sows that farrowed unsupervised overnight, colostrum samples were collected as soon as possible the following morning. Between 6 and 24 hours after birth, piglets were handled away from the sow for the recording of sex, birth weight and crown‒rump length and the assigning of an ear tag for identification. Colostrum samples (approximately 5 mL) were collected into large rimmed plastic beakers in one new container per sow (50 mL; ThermoFisher Scientific) before approximately 3–5 mL was decanted into a cryotube and transferred immediately to a storage freezer at ‒80°C.

### Colostrum sample analysis

Prior to analysis, colostrum samples were allowed to defrost at room temperature (20‒25°C) and were then vortexed for approximately 5‒10 seconds to ensure that the sample was thoroughly mixed. The colostrum IgG content was first estimated using a digital Brix refractometer (HI‐96801, Hanna Instruments). The Brix refractometer was calibrated using distilled water before each batch of samples (*n* = 20‒50) or when the laboratory temperature fluctuated by 5°C or more. One to two drops of undiluted colostrum were placed on the refractometer prism, and the percentage of IgG in the sample was estimated. The refractometer prism was cleaned before and after every sample using 70% ethanol to remove all residue. Each sample was analysed in duplicate consecutively, and the mean of both samples was used as the final Brix percentage estimate.

RID was also used to measure IgG concentration (Triple J Farms). Following the manufacturer's instructions, 5 µL of undiluted colostrum was aliquoted into each well of the test plate. The plates were incubated for 24 hours at room temperature in a moist chamber, and the zone of diffusion radius for each sample was measured using hand‐held digital Vernier callipers (IP54 water‐resistant Louisware electronic, accuracy 0.01 mm, range 0–150 mm). Each sample was tested in duplicate, and the final IgG concentration of each sample was estimated as the mean of both replicates.

### Statistical analysis

All the statistical analysis was completed using STATA (Stata Corp., version 18). The normality of the distributions of both RID (g/L) and Brix (%) measurements were assessed by plotting histograms and using Shapiro‒Wilk statistics (Shapiro‒Wilk <0.05 indicates normality). Descriptive statistics and Pearson's correlation coefficients were calculated for colostrum RID and Brix measurements.

A ROC curve was created to determine the optimal threshold to accurately predict poor colostrum quality using Brix, using RID as the reference standard. Based on other studies,^29^ colostrum was defined as inadequate when the IgG concentrations assessed by RID were less than 50 g/L. The Youden statistic^30^ was estimated to predict an accurate Brix threshold (i.e., empirical optimal cut‐off point) based on the sum of the sensitivity (Se) and specificity (Sp), with equal weight given to both false‐positive and false‐negative results.

The Se, Sp, positive likelihood ratio, negative likelihood ratio and accuracy of Brix measurement were estimated. The positive likelihood ratio was calculated as $\frac{\mathrm{Se}}{1-\mathrm{Sp}}$; the negative likelihood ratio as $\frac{1-\mathrm{Se}}{\mathrm{Sp}}$; and the accuracy as $\frac{\mathrm{true}\;\mathrm{positives}+\mathrm{true}\;\mathrm{negatives}}{\mathrm{total}\;\mathrm{number}\;\mathrm{of}\;\mathrm{samples}}$.

## RESULTS

Descriptive statistics for IgG concentrations and Brix measurements for all sows are shown in Table 1.

**TABLE 1:** Descriptive statistics for radial immunodiffusion (RID) and Brix refractometry measurements of colostrum from 87 sows in a single Scottish pig unit between September 2022 and July 2024

	Mean	SD	Minimum	Maximum	Median	IQR
Brix (%)	20.13	3.78	10.80	28.05	19.95	4.30
RID (g/L)	45.11	18.75	2.57	86.35	48.77	21.69

Abbreviation: IQR, interquartile range; SD, standard deviation.

**FIGURE 1:**
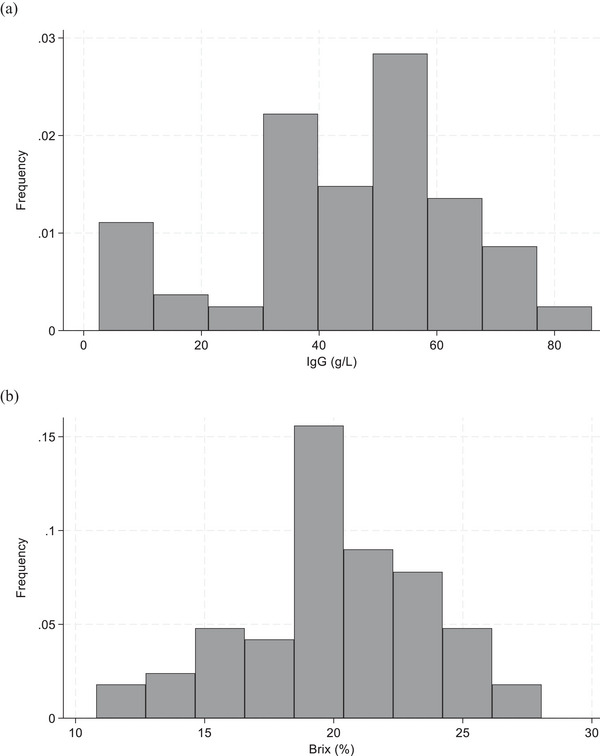
Frequency distribution of (a) concentrations of immunoglobulin G (IgG) measured by radial immunodiffusion, and (b) Brix refractometry measurements in colostrum samples collected from 87 sows on a single pig unit between September 2022 and September 2024

**FIGURE 2:**
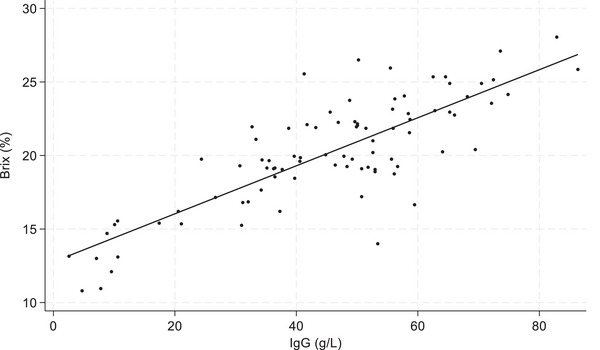
Scatter plot showing the relationship between concentration of immunoglobulin G (IgG) measured using radial immunodiffusion (g/L) and Brix digital refractometer measurement (%) in colostrum from 87 sows collected between September 2022 and September 2024

Colostrum samples from 40 of the 87 sows (45.98% of all samples) were below the colostrum quality threshold of 50 g/L. The distributions of IgG concentration and Brix percentage are shown in Figure 1. Shapiro‒Wilk tests indicated that while the Brix percentage measurements were normally distributed (Shapiro‒Wilk, *p* = 0.22), the RID IgG concentrations were not (Shapiro‒Wilk, *p* < 0.05). Multiple transformations did not improve the distribution, so the decision was made to use the untransformed values. Figure 2 shows the relationship between colostrum IgG concentration and Brix measurement. Pearson's correlation coefficient revealed a strong correlation of 0.81 (*p* < 0.001) between Brix percentage and RID IgG concentration.

**FIGURE 3:**
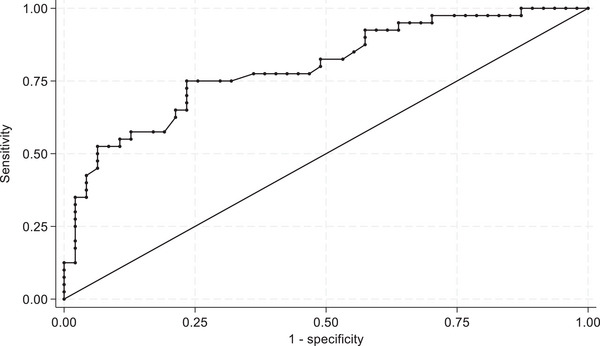
Receiver operating characteristic curve used to determine optimal cut‐off points for diagnosing poor colostrum quality (defined as immunoglobulin G concentration <50.0 g/L) using a Brix digital refractometer in samples collected from 87 sows on a single pig unit between September 2022 and August 2024

Figure 3 shows the ROC curve used to determine the optimal Brix colostrum threshold. The AUC describes the ability of a diagnostic test to differentiate between diseased and non‐diseased animals (i.e., good‐ or poor‐quality colostrum). The AUC in the present study was 0.80 for a Brix threshold of 22.00%, indicating that the Brix refractometry analysis is an accurate diagnostic test (AUC = 1 indicates a perfect diagnostic test). When the RID IgG threshold of less than 50 g/L was used, ROC analysis defined an optimal Brix threshold as 20.05%. At the empirical optimal cut‐off point (Brix cut‐off point = 20.05%) defined by Youden's statistic,^30^ Se and Sp were 0.75 and 0.75, respectively, while the AUC at that point was 0.76. Table 2 shows the diagnostic test performance of the Brix refractometer (Se, Sp, positive likelihood ratio, negative likelihood ratio and accuracy) at the published cut‐off point of 22.00% and the newly defined cut‐off point of 20.05% (using RID IgG <50 g/L as the threshold for poor IgG concentration).

**TABLE 2:** Diagnostic test results for Brix refractometry when used to predict colostrum quality (adequate quality defined as immunoglobulin G concentrations <50 g/L in colostrum measured by radial immunodiffusion [RID] reference test) in sows (*n* = 87) on a single Scottish pig unit

RID threshold (g/L)	Brix threshold (%)	Se		Sp		PPV		LR+		NPV		LR‒		Accuracy	
		*n*	%	*n*	%	*n*	%	Se/1 ‒ Sp	Ratio	*n*	%	1 ‒ Se/Sp	Ratio	*n*	%
50	22.00	41/47	87.23	23/40	57.50	41/58	70.69	0.87/0.43	2.02	23/29	79.31	0.13/0.56	0.23	64/87	73.56
	20.05	35/47	74.46	30/40	75.00	35/45	77.77	0.74/0.25	2.96	30/42	71.42	0.26/0.75	0.34	65/87	74.71

*Note*: Cut‐off points were derived from published data (22%) or based on receiver operating characteristic curve analysis (20.05%).

Abbreviations: LR+, positive likelihood ratio; LR‒, negative likelihood ratio; NPV, negative predictive value; PPV, positive predictive value; Se, sensitivity; Sp, specificity.

## DISCUSSION

The present study compared the Brix refractometer to the RID reference test to define an appropriate threshold for porcine colostrum from a single Scottish pig unit. Colostrum Brix measurements (mean = 20.13, standard deviation [SD] = 3.78) were slightly lower than those reported elsewhere (mean = 25.0, SD = 0.29^27^; mean = 24.1, SD = 2.65^14^). This is most likely due to the timing of colostrum sample collection, since samples in other studies were collected within 3 hours of farrowing^5^ or between 9 hours pre‐farrowing and 14 hours post‐farrowing.^14^ Balzani et al. reported that the colostrum IgG concentration was highest in samples collected up to 9 hours before farrowing (mean Brix = 24.8%) and lower when samples were collected after 4 hours post‐farrowing (mean Brix = 22.6%).^14^ Only 32 of the 87 samples in the present study were collected within 6 hours of the estimated onset of farrowing, and 85 of the 87 samples were collected after all the piglets had been born, meaning that the majority of piglets would already have suckled from the dam. The majority of samples in the present study were, therefore, not ‘first milk’, and this will have contributed to the non‐normal distribution of IgG measurements using RID. It is well documented that the IgG concentration declines with time post‐parturition and with increasing number of ‘milkings’ post‐parturition.^30^ Nevertheless, since the purpose of the present study was to validate the use of a Brix refractometer for its diagnostic usefulness in estimating on‐farm colostrum quality rather than for calculating a prevalence estimate for poor colostrum quality, it was important that variation in colostrum IgG concentration existed in the sample dataset rather than samples being strictly ‘first milk’. Future studies may focus on the collection of colostrum as close to farrowing as possible to define the prevalence of low first‐milking colostrum quality. However, it should be noted that litter size and duration of farrowing may influence the optimum timeframe for colostrum collection post‐farrowing.

Oliviero reported average sow farrowing times ranging between 212 and 301 minutes (3.53‒5.02 hours), where the average litter size was 12.7 and duration of farrowing was defined as the time in minutes between the first and last piglets born.^10^ Since this study was performed, the average litter size and farrowing duration in commercial pig herds have increased. The same author reviewed 20 studies reporting increases in litter size from approximately 10 piglets in 1990 to close to 20 piglets in 2019 and increases in duration of farrowing from 1.5–2 hours to 7–8 hours.^31^ In terms of colostrum quality, this extended farrowing duration has implications for piglets born later in the birth order, with those born earlier in the litter having access to the true first milk produced by the dam. Indeed, Quesnel et al. identified time of birth after the onset of parturition, as well as birth weight and rectal temperature 1 hour after birth, as significant explanatory variables for variable colostrum intake in piglets.^6^ While it is unrealistic to expect stock people in farrowing units to analyse multiple colostrum samples from a sow using a Brix refractometer at various time points during farrowing, it could be suggested that the optimal time of sampling should be mid‐way through farrowing (e.g., after the birth of the sixth or seventh piglet, depending on typical litter sizes for the herd) to get a more realistic estimation of the mean colostrum quality available to the litter as a whole.

In the present study, a strong positive correlation (0.81; *p* < 0.001) was demonstrated between colostrum IgG concentration measured using RID and the Brix refractometer. It is acknowledged that correlation is not a measure of test agreement; however, agreement statistics are challenging where tests are measured at different scales. The ROC curve defined a Brix threshold of 20.05% for colostrum, using an RID threshold of 50 g/L extrapolated from other species. The specificities of the Brix assessment of IgG concentration in colostrum using Brix thresholds of 20.05% and 22.00% were 75.00% and 57.50%, respectively. The 22.0% threshold is the industry standard used elsewhere in dairy cattle^25^ and sheep,^32^ with the aim of maximising specificity and reducing the risk of false‐positive results. However, in the present study, the specificity of the Brix test at a 20.05% threshold (75.00%) was higher than that at a 22.00% threshold (57.50%). This indicates that false‐positive results may be more frequent when the 22.00% Brix threshold is used.

Brix measurements should primarily be used as a screening test for IgG concentration in colostrum rather than a diagnostic tool for individual samples. Screening tests generally maximise diagnostic test specificity to result in fewer false‐positive results. Fewer false‐positive results (i.e., colostrum misclassified as high IgG concentration when in fact it is low IgG concentration) reduces the risk of poor‐quality colostrum intake by piglets, thereby reducing possible FTPI and subsequent health and productivity deficits.

Positive and negative predictive values are driven by the prevalence of the disease (in this case, poor‐quality colostrum; IgG < 50 g/L) and are therefore only relevant within the test population or a population with identical disease prevalence as the study population. For example, an excellent test may have a poor positive predictive value if the prevalence of the disease is very low. To overcome this, positive and negative likelihood ratios were estimated in the present study. Likelihood ratios do not depend on the prevalence of the disease, so outcomes from a test population can be applied to other populations.

### Study limitations

All samples in the present study were collected from a single research pig unit, which may influence the external validity of the results. Approximately 2 weeks before analysis, samples were moved and stored in a freezer at ‒20°C until analysis, which may have compromised sample quality; however, every effort was made to preserve sample quality using low‐temperature storage.

While this study would have benefitted from the acquisition of piglet serum samples to investigate relationships between porcine colostrum IgG and piglet serum IgG, this was not possible as this was not the aim of the larger study from which these samples were attained. This work was also retrospective and opportunistic, as the sows and gilts included in this study were enrolled in a primary trial exploring farrowing and early piglet life.

## CONCLUSION

The results of the present study indicate that the Brix refractometer can be used as a fast and accurate pen‐side tool for estimating the IgG concentration in porcine colostrum, with a strong positive correlation between RID reference standard values and Brix measurements. Brix test specificity was maximised by reducing the threshold of 22.00% (extrapolated from other species) to 20.05% to minimise the risk of poor‐quality colostrum being fed to piglets.

## AUTHOR CONTRIBUTIONS

Áine C. O'Brien and Katharine S. Denholm conceived and presented the idea for this study. Katharine S. Denholm developed the theory and performed the computations. Emma M. Baxter and Marianne Farish verified the analytical methods. All the authors discussed the results and contributed to the final manuscript.

## CONFLICT OF INTEREST STATEMENT

None of the authors has any financial or personal relationships that could inappropriately influence or bias the content.

## ETHICS STATEMENT

This study was part of a larger programme of work that was reviewed and approved by SRUC's Animal Ethics Review Board (AE14‐2022).

## Data Availability

The data that support the findings of this study are openly available from the University of Glasgow (https://doi.org/10.5525/gla.researchdata.2004).
